# Radiology for medical students (1925–2018): an overview

**DOI:** 10.1259/bjro.20190050

**Published:** 2020-02-04

**Authors:** Cindy Chew, Paul Cannon, Patrick J O'Dwyer

**Affiliations:** 1 Department of Radiology, University Hospital Hairmyres, East Kilbride, Scotland; 2 School of Medicine, Dentistry and Nursing University of Glasgow, Glasgow, Scotland; 3 Medical, Veterinary and Life Sciences, University Library University of Glasgow, Glasgow, Scotland; 4 Emeritus Professor Gastrointestinal Surgery, University of Glasgow, Glasgow, Scotland

## Abstract

**Objective:**

Radiology has been espoused as an excellent tool for educating medical students since 1925. Advances in technology and PACS mean it has never been easier to demonstrate living anatomy and clinical pathology in exquisite detail to students. The aim of this study was to provide an overview of radiologic publications related to teaching medical students and its evolution through time.

**Methods:**

A literature search was performed from inception to November 2018. The search strategies used both text words and relevant indexing related to “radiology”, “medical students” and “curriculum”.

**Results:**

3589 records were identified of which 377 were included. There was a 100 fold increase in rate of publication over time—most were expository or surveys (60%), with few truly experimental articles. Radiology was used in clinical teaching (67%) and anatomy (33%). Almost half of radiologic anatomy teaching was conducted without the input of a Radiologist. Compulsory clinical clerkships/blocks in radiology was offered infrequently (35%). Female first authorship had increased in the last decade (47%).

**Conclusion:**

There is a significant increase in articles published on the role of radiology in medical student teaching in the last decade. Research in this area is required in order to investigate the role of radiology in improving the modern medical students’ education.

## Introduction

Radiology has come a long way since Roentgen first discovered X-ray in 1895.^1^ Thanks largely to technological advancements in CT, MRI, positron emission tomography (PET)/CT and ultrasound, radiology is now a vital part of clinical medicine, central in the diagnostic process as well as providing therapeutic options for patients. Almost 100 years of articles chart the evolution of the role of radiology in teaching medical students—from editorials by the early professors of radiology (1925–1950) extolling the untapped resource of radiologists to teach anatomy and clinical medicine, through to the calls for compulsory lectures, elective then compulsory “clerkships” (1980s). The 1990s saw articles on adopting technological advances to incorporate radiology easily into the curriculum. Radiologists are perfectly placed to meet three of the four core learning profiles in pedagogy of learning: the Auditory, Visual and Reading/Writing learner. Twenty-first century Radiology teaching is focused on defining a core syllabus, assessment, appropriateness of investigations and web-based online interactive teaching and learning models.

The aim of this study was to provide an overview of radiologic publications related to teaching medical students and its evolution through time.

## Methods

Institutional Board Review was not required as this study involved the review of published literature.

MEDLINE (Ovid), Embase (Ovid), the Cochrane Database of Systematic Reviews and CENTRAL (Wiley Interscience) and the Education Resources Information Centre and British Education Index (EBSCOhost) databases were searched from inception to November 2018. No restrictions was placed on publication date within these databases. This review included English language studies only.

A search strategy was developed in collaboration with the University Subject Librarian using a combination of subject heads and text words related to “Radiology,” “medical students” and “curriculum”. The search output was reviewed to ensure the strategy detected relevant references. The search was deliberately comprehensive to allow as many potentially eligible articles as possible to be identified.

All studies that described the use of radiology or radiological images to teach medical students were included. There was no requirement for the studies to have a comparator intervention. Studies were included if they included one of the following: radiology, medical student teaching, radiological images used to teach (X-ray, CT, MRI, ultrasound). Articles not involving medical students or involved students of mixed disciplines, articles on education only (not involving radiology) and articles regarding recruiting medical students into radiology were excluded.

Two reviewers independently scanned the titles and abstracts of all references downloaded onto the bibliographic software (EndNoteX9^®^) to identify potentially relevant articles. For all references that met the inclusion criteria a copy of the full article was retrieved. References that did not meet the inclusion criteria were coded according to the exclusion criteria. Any disagreement at screening or retrieval stage was resolved by discussion; a third reviewer was available to be consulted but was never used. Reviewer bias was mitigated by having two non-radiologists involved in the screening and review process.

Data were collected for each included study using a pre-designed data extraction form. The analysis of the articles followed that performed by Collins et al^2^ in their review, which was modified from Calhoun et al,^3^ namely (a) source of publication (b) year published (c) degree (s) of first author (d) number of authors (e) type of article (editorial, expository, survey, correlational, experimental) (f) topic of article. Articles’ topic /subject of interest were analysed: philosophical or political, program evaluation, examinations, program description and technology. Detailed description of each is included in Supplementary Material 1. Our study included additional categories for evaluation: (1) first author’s gender; (2) origin of the articles; (3) the setting of teaching (preclinical or clinical); (4) whether radiologists were involved when using radiologic images to teach anatomy and (5) whether radiologists were involved in teaching students ultrasound. Data were summarised according to outcomes of interest. Results were tabulated and represent a comprehensive narrative review of the literature.^4^

## Results

**Figure 1.**
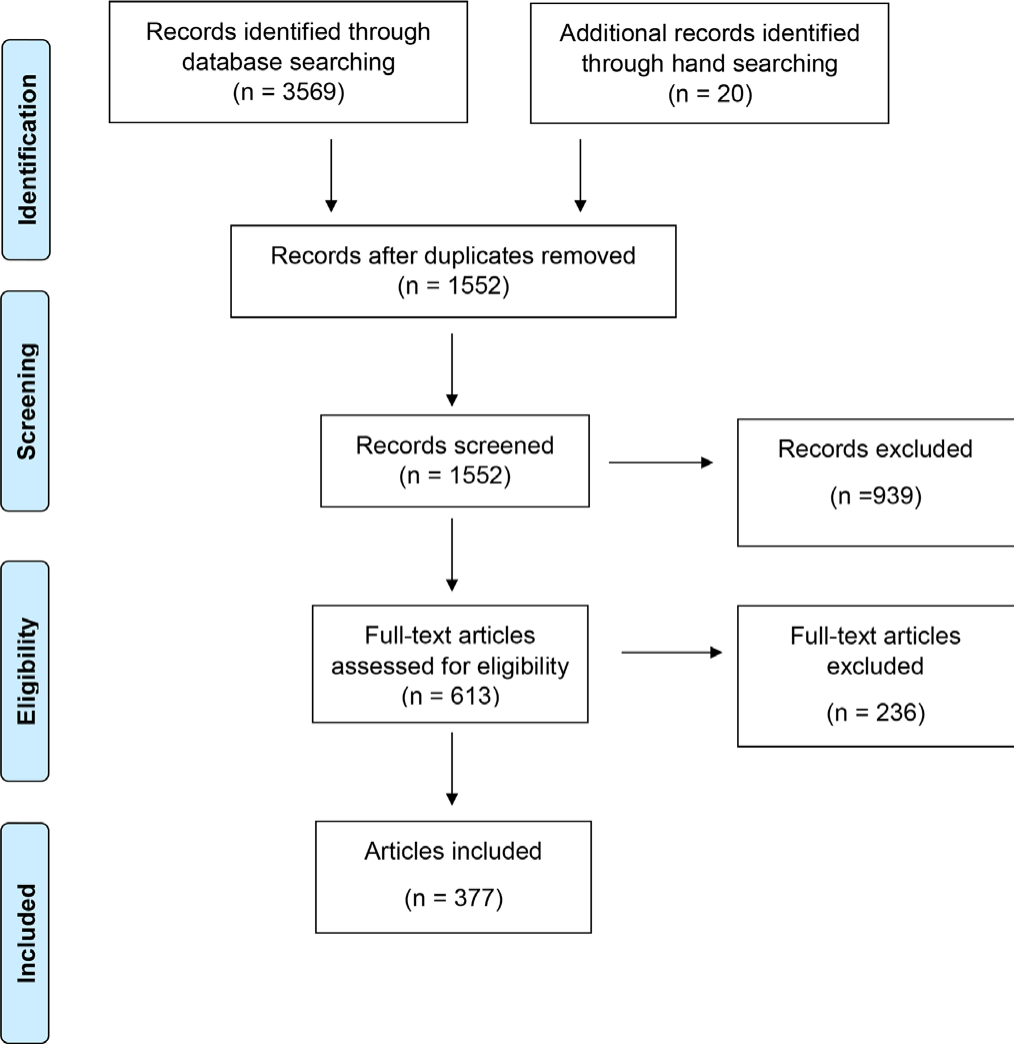
Flow diagram of records.

3589 records were identified. 1552 unique abstracts were screened for inclusion. 613 full text articles were assessed, of which 377 were included for evaluation. This included 20 articles identified by hand searching. The flow diagram is demonstrated in Figure 1. Included articles are listed in Supplementary Material 2.

## Articles published over time

The total number of articles published related to radiology in medical student education included in this study is 377. The first paper describing the use of radiology in teaching medical students was published in 1925–30 years after Roentgen discovered X-ray in 1895.^5^ First paper to describe the use of radiologic images to teach anatomy was published 2 years later—by a radiologist.^6^ The rate of publication per year was 0.2 during the early period of 1950–1959 compared to 20.3 in the most recent 9 years. This is an increase of 100-fold (Table 1).

**Table 1.** Rate of articles published over time

Period (years)	Articles published	Articles published per year
1950–1959	2	0.2
1960–1969	6	0.6
1970–1979	29	2.9
1980–1989	33	3.3
1990–1999	36	3.6
2000–2009	84	8.4
2010–2018a	183	20.3

a Note : Time interval 9 years instead of 10 years.

## Source of publications

Articles on the subject of radiology in medical student education are published in 98 journals. Collins et al noted a change in publication forum from *Investigative Radiology* to *Academic Radiology* when the latter began publishing in 1994.^2^ *Academic Radiology*, the official journal of the Association of University Radiologist, continued to dominate the publishing scene with 90 articles (24%). The next largest number of articles was published in the *Journal of American College of Radiology* - with a third as many articles (*n* = 29; 8%). Together, the top 10 journals published 64% (*n* = 242) of the articles relating to Radiology in undergraduate medical education (Table 2).

**Table 2.** Top 10 journals for publications (*N* = 242)

Journal	Number of articles published (% of total)
Academic Radiology	90 (24%)
Journal of American College of Radiology	29 (8%)
Investigative Radiology	29 (8%)
Anatomical Science Education	19 (5%)
Radiology	18 (5%)
Clinical Radiology	15 (4%)
European Journal of Radiology	15 (4%)
American Journal of Roentgenology	13 (3%)
Medical Education	7 (2%)
Australasian Radiology	7 (2%)

## Origin of articles published

The vast majority of articles on radiology in medical student education originated from the USA (*n* = 225, 60%). Europe produced 88 articles (23%) on this subject and Canada 20 (5%) (Table 3). Within Europe, the United Kingdom contributed the largest number of articles (*n* = 36 of 88, 41%), with Germany (*n* = 14; 16%) and The Netherlands (*n* = 12; 14%) next most active in the field.

**Table 3.** Origin of articles

Country	Number of articles (%)
USA	225 (60%)
Europe	88 (23%)
Canada	20 (5%)
Australia/New Zealand	14 (4%)
Middle East	12 (3%)
Asia	11 (3%)
Africa	7 (2%)

## Articles: type, topic, article length

**Figure 2.**
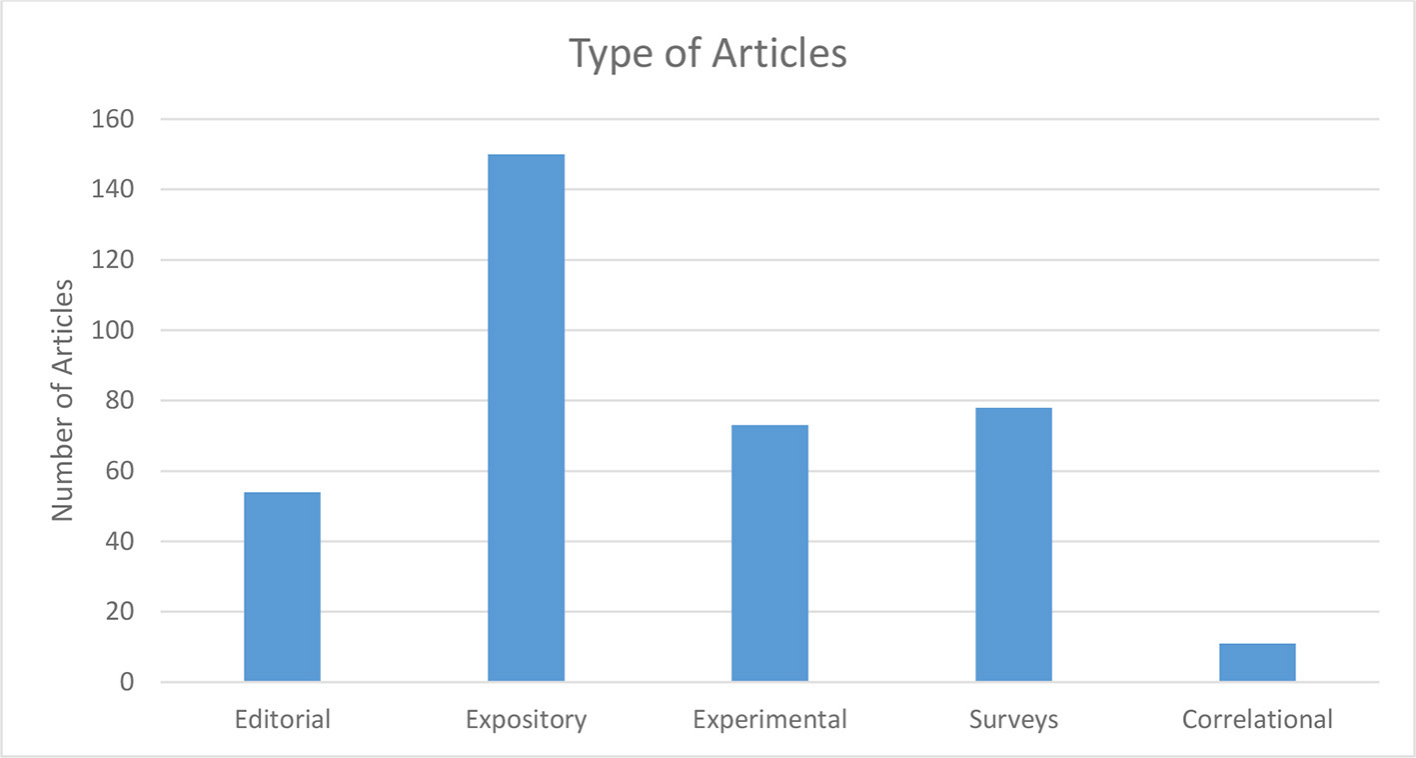
Types of articles.

**Figure 3.**
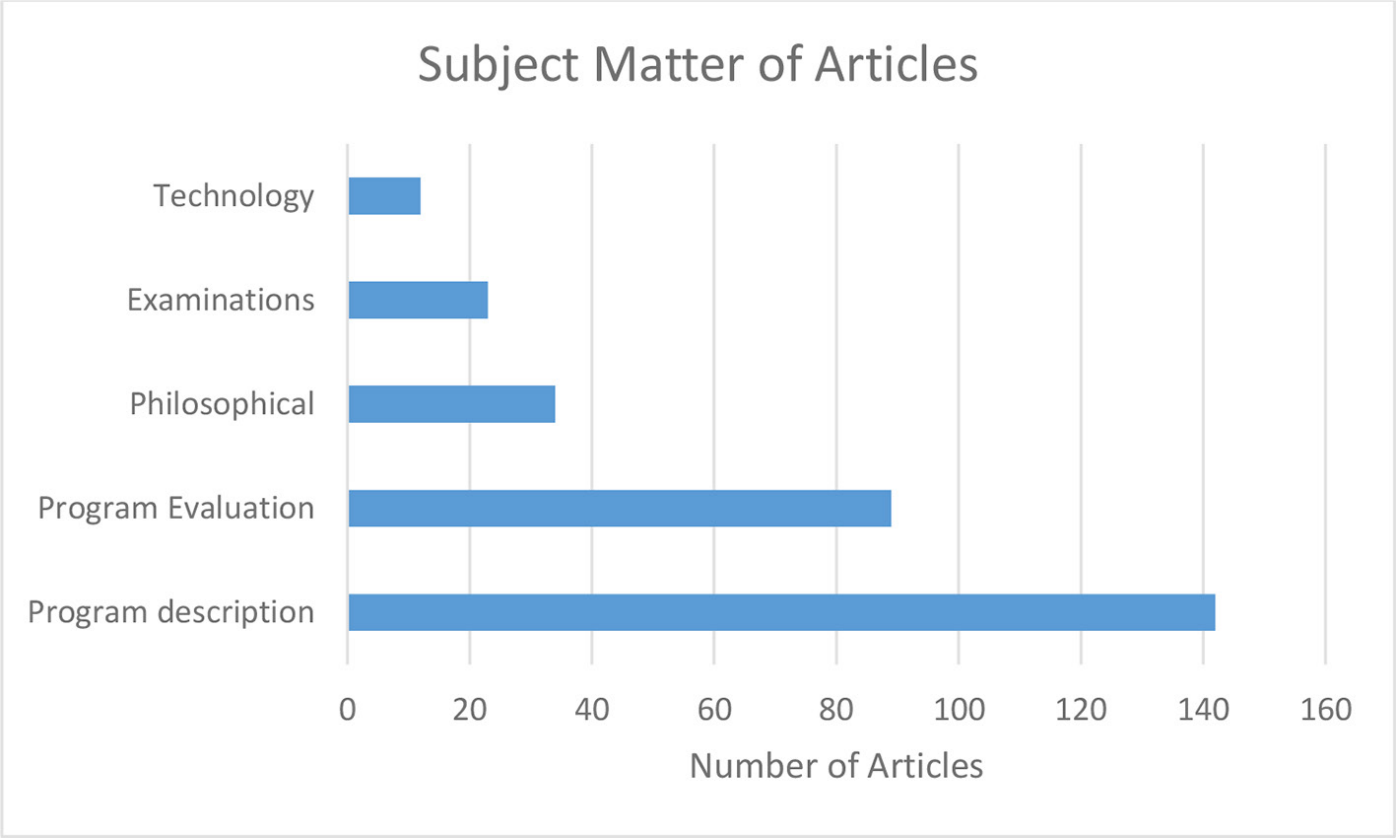
Topic of articles.

Expository articles were the most common type of articles, contributing 39% of papers on the subject, with experimental papers and surveys combining to make up another 39% (Figure 2). Only 13% (*n* = 10) of the experimental papers were truly experimental (adequate control for internal invalidity, randomly assigned test and controlled groups, pre-test post-test control group design).^2^ However the topics studied were diverse, with small student numbers and of poor quality. Program description and evaluation made up 60% of article topics (37 and 23% respectively, Figure 3). The median number of pages per article was 6 (range 1–16, interquartile range: 4).

## Authorship

Our study, focusing solely on radiology in medical student education, demonstrated a trend towards an increased number of authors per article over time. Most papers during the early years (1950–1979) were written by one person (25/39, median 1, range 1–4). During 1980–2009, the most common author number was 3 (range 1–11), while in the last 9 years it was 4 (range 1–14, Table 4). First author was most often medically qualified (84%) and of those 70% were radiologists.

**Table 4.** Median number of author per publication over time

Period	Median number of authors	Range
1950–1959	1	1
1960–1969	1	one to 3
1970–1979	1	one to 4
1980–1989	3	one to 7
1990–1999	3	one to 10
2000–2009	3	one to 11
2010–2018a	4	one to 14

a Note : Period covered 9 years instead of 10 years.

**Figure 4.**
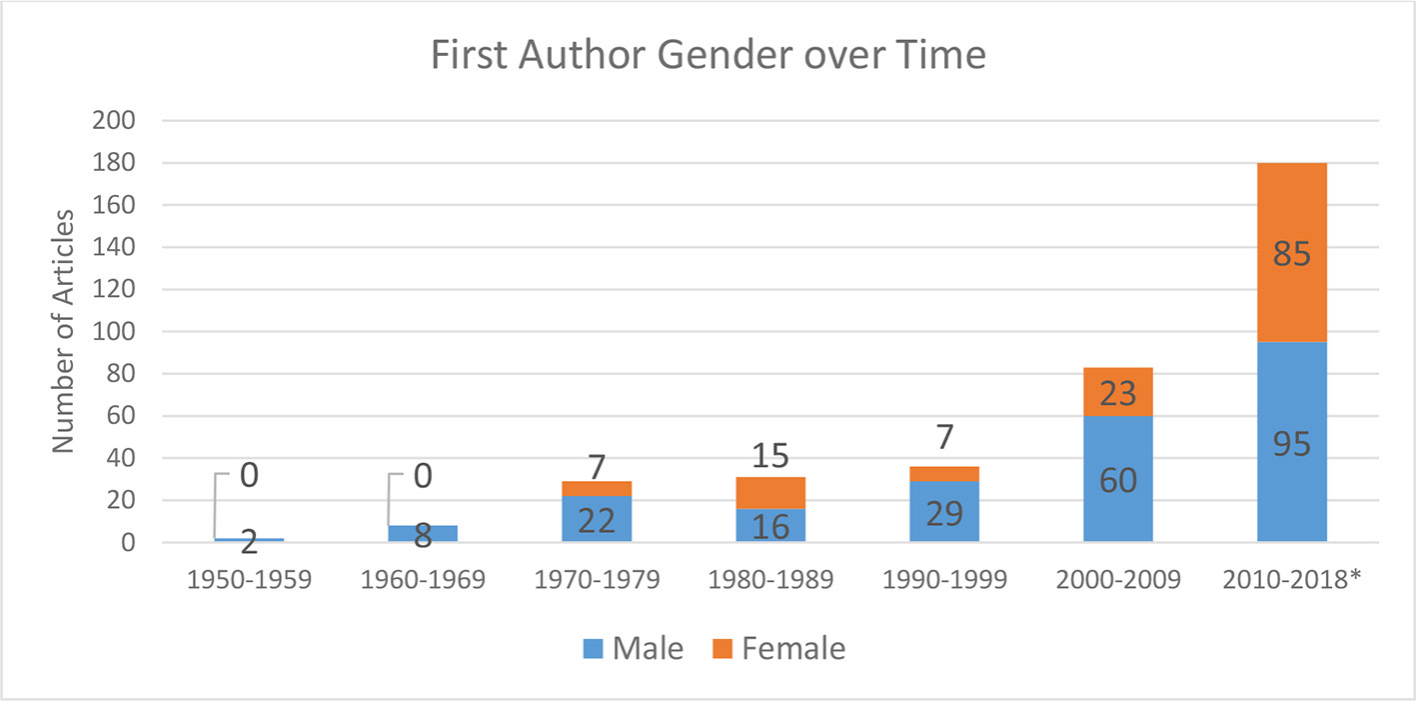
First author gender over time.

The majority of first authors were male (62%). There was an increasing number of female first authors from 1970s, with females making up 47% of first authors in the most recent 9 years (Figure 4).

## Setting of teaching: clinical v pre-clinical

**Figure 5.**
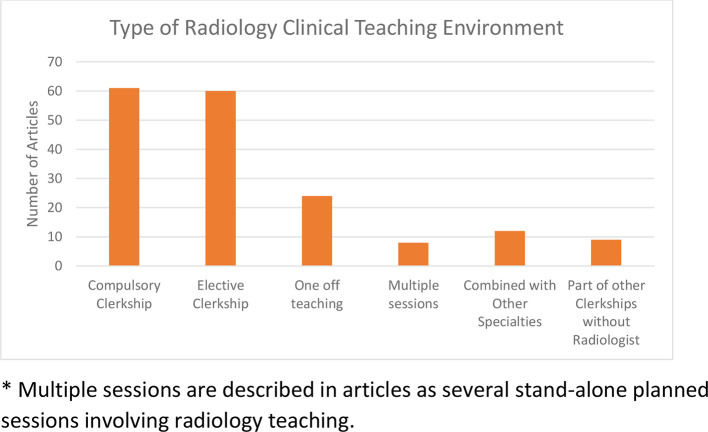
Clinical teaching setting.

**Figure 6.**
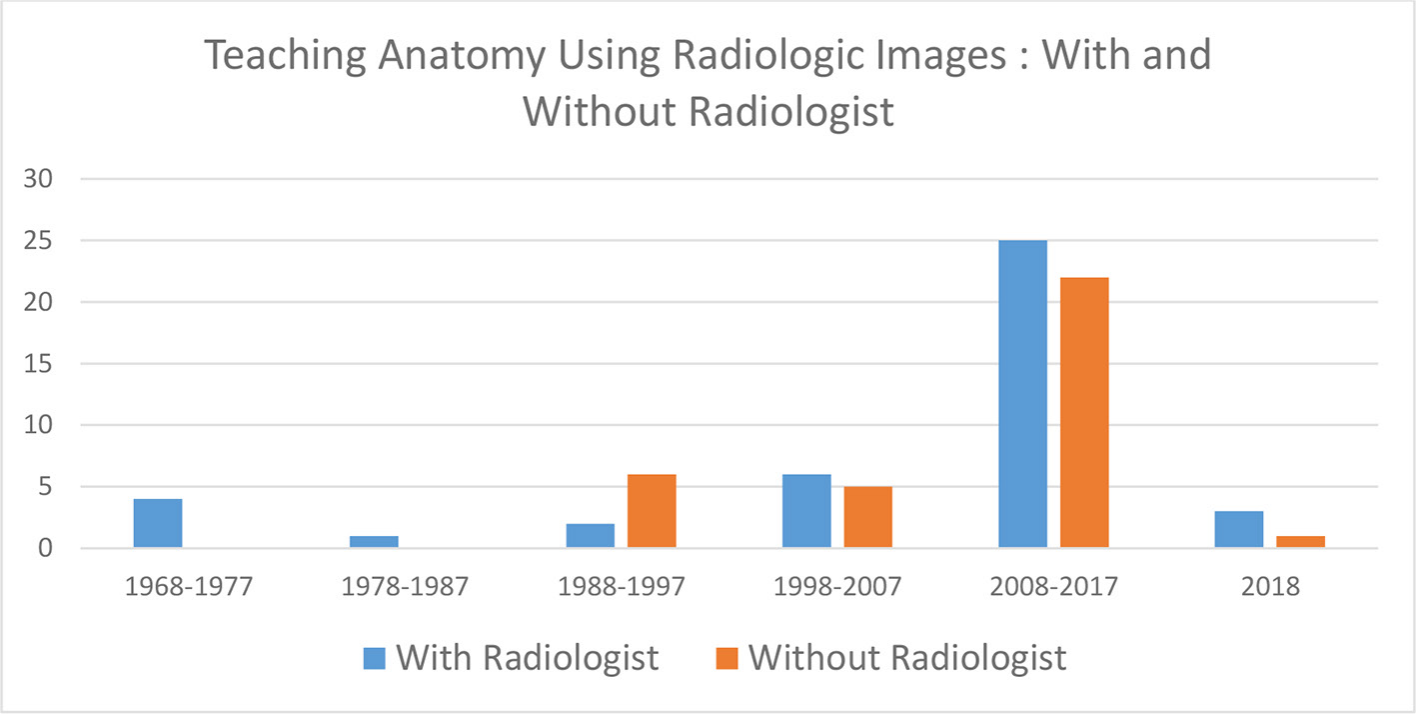
Anatomy teaching—with and without radiologist input over time

Over two-thirds of articles were concerned with education during the clinical phase of learning (192/286). Universities offered compulsory radiology clerkships (clinical blocks) infrequently (35%; Figure 5). Radiology clerkships/blocks ranged from 1 to 6 weeks. Pre-clinical medical education is mostly concerned with anatomy. Overall involvement of radiologists during these sessions was 57%. A persistent trend in anatomy education using radiologic Images without radiologists involvement was observed from 1990 onwards (Figure 6).

## Ultrasound

Point of care ultrasound is gaining clinical use. Only 29 articles related to teaching ultrasound to medical students were identified in our study (8%). Almost a third of the articles described teaching ultrasound to medical students without any radiologist input.

## Discussion

We have demonstrated a large 100-fold rise in papers espousing the merit of radiology teaching in the undergraduate medical curriculum over the last 70 years. Despite this, the field remains composed mostly of expositories and program description articles—describing how radiology is/can/should be taught—often against a back drop of limited university or departmental support and resource.^7–9^ The ever increasing volume of clinical work makes teaching an even more challenging task. The USA is most prolific in this field of study, yet only 25% of American universities include Radiology in their curriculum.^10^ The Alliance of Medical Student Educators in Radiology, within the Association of University Radiologists, has undertaken a significant amount of work to address this. ASMER has developed a National Medical Student Curriculum to identify core competencies in Radiology, listing learning objectives, as well as establishing a nationally deployable, validated, web-based computer mark-able radiology examination.^11^ They are currently working on an ultrasound curriculum for medical students. All these indicate a strong willingness, at least for now, of radiologists to teach medical students. It also highlights persistent barriers to consistent radiology/radiologists inclusion into the medical curriculum, perhaps related to balancing between University/Departmental funding streams and workload.^8^

In Europe, the situation is as diverse. An international survey of radiologists coordinated by the European Society of Radiologists shows most European undergraduate medical education span 6 years (range 4–8 years), with 92% reporting inclusion of radiology as part of the formal curriculum (Denmark and United Kingdom excepted).^12,13^ Half of the respondents report radiology is an independent discipline with its own examination. The median total hours spent on radiology teaching is 76 h (range 19–212 h). A follow-up survey of 93 (430 polled) institutions throughout Europe records a median 59 or 66 h devoted to radiology, depending on whether the institution adopts a modern (problem-based) or conventional medical curriculum, with between 8 (conventional curriculum) to 15 (modern curriculum) members of staff involved in teaching.^14^ The majority of time is spent on clinical medicine based around clerkships/blocks. Radiology clerkships are compulsory in 55% of institutions with modern medical curriculum lasting 4.4 weeks compared to 59% in those adopting conventional curriculum (lasting 5.1 weeks). Specific radiology curriculum in the undergraduate medical education has been published by the European Radiology Society.^15^

In the United Kingdom, the Royal College of Radiologists also publishes its own undergraduate radiology curriculum, mapped to the General Medical Council’s “Outcomes for Graduates” document (2015).^16^ The UK undergraduate medical education is predominantly a 5 year course, and UK reports total hours dedicated to radiology teaching within the median range, in the 2011 European Society of Radiologists survey (44–116 h).^12^ This is an improvement from a 1981 report stating academic department medical student teaching occurred only in Bristol, Cambridge, Cardiff, Edinburgh, Liverpool, Oxford and Nottingham, with none in London.^17^ Clerkships/clinical blocks in radiology are uncommon and if present would be in the form of an elective. No national radiology syllabus exist in Canada, Australia or New Zealand and a study reports 276 h dedicated to Radiology in New Zealand, 165 h in USA compared to 85 h in Australia. The difference is attributed to University Chairs in Radiology.^18^

The key role of radiology in undergraduate medical education, is not so much to produce miniradiologists, as to provide a solid, often visual framework upon which medicine can be taught to students.^19,20^ Almost every pathological condition a student needs to learn has a radiologic manifestation to diagnose it. In addition, radiology is a powerful tool to teach clinical reasoning—going through the patient’s history, generating a differential diagnosis, and deciding which is the best (most accurate and cost efficient) test to confirm the diagnosis to commence treatment.^21^ Surveys demonstrate medical students feel poorly prepared for clinical practice with regards to radiological examinations, and clinical leaders expressing the need for more radiology input into medical student education to prepare them for clinical practice.^22^

A trend exists where clinicians (30%) and anatomists (43%) appear quite comfortable teaching medical students on radiological images without the input of a radiologist.^23–26^ While this attitude could be understood in the past, where radiology consisted mainly of plain radiographic images, this is no longer the case today. CT is the acknowledged work horse of the clinical diagnostic process and together with MRI, are sophisticated examinations producing exquisitely detailed but complex images which takes a radiologist five or more years to be competent to read.

First author gender is a specific outcome measure in this study. Overall, more males than females first authors is identified. Although females make up 27, 39 and 44% of first authors in articles from UK, USA and Europe (*p* > 0.05), there is a healthy trend towards almost equal gender contribution to the literature in the last 9 years. While this may be expected against a background of increasing proportion of female medical students—55% in the UK (2016) and 50% in USA (2017)—this is particularly heartening given only 27% of radiology residents (trainee radiologists) are females in the USA compared to 46% in the UK (2015).^27–30^

The future of medical student teaching by radiology is juxtaposed starkly against the ever increasing incessant demands on the specialty. There are (international) workforce issues—75% of radiology clinical directors in the UK report insufficient radiologists to deliver safe and effective patient care.^31^ However, technological advances mean we can still provide interactive, authentic immersive teaching using a variety of virtual and online tool with limited manpower.^32,33^ Standardised validated web-based self-marking, nationally deployable examinations are in use in the USA, using exquisite whole body CT/MRI images of real patients.^11^ The future trend is likely the migration of radiology education into the social media sphere.^34,35^ Best pedagogic practice has been adopted and continue to be shared—small group teaching, clinical seminars, case presentations, structured and self-learning, defined learning objective, assessment in radiology and flipped classrooms.^36^ These serve to promote active, experiential and authentic learning. Thematically, the emphasis is distinctly one of teaching medicine and the appropriateness of radiological examinations—“the right test for the right patient at the right time.”^37^

One of the limitations of this study is that it only included articles published in the English language. Another drawback is some articles could be missed if “radiology,” “medical students,” “curriculum” were not included as key words. Despite such limitations, this article remains the most extensive overview of radiology for medical students in the literature.

## Conclusion

There has been a significant increase in articles published on radiology in the teaching of medical students in the last decade. Most of the articles remain expositories and surveys—few are truly experimental. There is a trend of non-radiologists teaching radiology—the appropriateness of this in the 21st century is questionable. Gender equality in first author has almost been achieved in this field over the last 9 years.

Quality research is required to investigate what role radiology has in improving medical education for medical students in the modern era.
